# *Trans*-Cinnamaldehyde and Eugenol Increase *Acinetobacter baumannii* Sensitivity to Beta-Lactam Antibiotics

**DOI:** 10.3389/fmicb.2018.01011

**Published:** 2018-05-23

**Authors:** Deepti P. Karumathil, Meera Surendran Nair, James Gaffney, Anup Kollanoor-Johny, Kumar Venkitanarayanan

**Affiliations:** ^1^Department of Animal Science, University of Connecticut, Storrs, CT, United States; ^2^College of Veterinary Medicine, Cornell University, Ithaca, NY, United States; ^3^Department of Animal Science, University of Minnesota, Saint Paul, MN, United States

**Keywords:** *A. baumannii*, *trans*-cinnamaldehyde, eugenol, antibiotic resistance

## Abstract

Multi-drug resistant (MDR) *Acinetobacter baumannii* is a major nosocomial pathogen causing a wide range of clinical conditions with significant mortality rates. *A. baumannii* strains are equipped with a multitude of antibiotic resistance mechanisms, rendering them resistant to most of the currently available antibiotics. Thus, there is a critical need to explore novel strategies for controlling antibiotic resistance in *A. baumannii*. This study investigated the efficacy of two food-grade, plant-derived antimicrobials (PDAs), namely *trans*-cinnamaldehyde (TC) and eugenol (EG) in decreasing *A. baumannii*’s resistance to seven β-lactam antibiotics, including ampicillin, methicillin, meropenem, penicillin, aztreonam, amoxicillin, and piperacillin. Two MDR *A. baumannii* isolates (ATCC 17978 and AB 251847) were separately cultured in tryptic soy broth (∼6 log CFU/ml) containing the minimum inhibitory concentration (MIC) of TC or EG with or without the MIC of each antibiotic at 37°C for 18 h. *A. baumannii* strains not exposed to the PDAs or antibiotics served as controls. Following incubation, *A. baumannii* counts were determined by broth dilution assay. In addition, the effect of PDAs on the permeability of outer membrane and efflux pumps in *A. baumannii* was measured. Further, the effect of TC and EG on the expression of *A. baumannii* genes encoding resistance to β-lactam antibiotics (*blaP*), efflux pumps (*adeABC*), and multi-drug resistant protein (*mdrp*) was studied using real-time quantitative PCR (RT-qPCR). The experiment was replicated three times with duplicate samples of each treatment and control. The results from broth dilution assay indicated that both TC and EG in combination with antibiotics increased the sensitivity of *A. baumannii* to all the tested antibiotics (*P* < 0.05). The two PDAs inhibited the function of *A. baumannii* efflux pump, (AdeABC), but did not increase the permeability of its outer membrane. Moreover, RT-qPCR data revealed that TC and EG down-regulated the expression of majority of the genes associated with β-lactam antibiotic resistance, especially *blaP* and *adeABC* (*P* < 0.05). The results suggest that TC and EG could potentially be used along with β-lactam antibiotics for controlling MDR *A. baumannii* infections; however, their clinical significance needs to be determined using *in vivo* studies.

## Introduction

*Acinetobacter baumannii* is a multi-drug resistant (MDR) Gram-negative, aerobic bacillus that has emerged as a major cause of nosocomial infections with mortality rates ranging from 34 to 61% (Karageorgopoulos and Falagas, 2008; Wieczorek et al., 2008; Esterly et al., 2011). In humans, MDR *A. baumannii* causes a wide-spectrum of clinical conditions, including pneumonia (Leung et al., 2005), blood-stream infections (Wisplinghoff et al., 2004; Centers for Disease Control and Prevention [CDC], 2004), meningitis (Metan et al., 2007), urinary tract infections (Sunenshine et al., 2007), and wound infections (Scott et al., 2007) In addition, reports of other manifestations such as endocarditis, peritonitis, and osteomyelitis associated with *A. baumannii* have been reported (Olut and Erkek, 2005; Menon et al., 2006) *A. baumannii* is ranked as one of the most common bacteria associated with intensive care units (Garnacho-Montero and Amaya-Villar, 2010; Ulu-Kilic et al., 2013), and is difficult to treat due to its resistance to most of the currently available antibiotics (Maragakis and Perl, 2008; Doi et al., 2009; Neonakis et al., 2011; Al Mobarak et al., 2014; Ellis et al., 2015) For example, carbapenems, which were once the antimicrobials of choice for treating *A. baumannii*, are no longer completely effective due to resistance development by the bacterium (Falagas et al., 2006; Bassetti et al., 2008; Abbott et al., 2013; Fonseca et al., 2013). Similarly, although polymyxins have been successfully used to treat *A. baumannii* infections, strains resistant to these drugs have appeared (Hernan et al., 2009; Lean et al., 2014; Pogue et al., 2015). In light of these reports, the Infectious Diseases Society of America ranked *A. baumannii* as one of the top priority, antibiotic-resistant pathogens to target due to its rapid propensity to develop drug resistance, and a limited choice of antibiotics available to treat infections caused by this bacterium (Talbot et al., 2006; Shlaes et al., 2013).

*A. baumannii* is considered to be multidrug resistant, if it exhibits resistance to more than three classes of antibiotics (Falagas et al., 2006). The resistance of *A. baumannii* to antibiotics has been attributed to multiple mechanisms, including reduced permeability of its outer membrane to antibiotics, constitutive expression of drug efflux pumps, and its ability to acquire and incorporate genetic elements such as plasmids, transposons, and integrons (Giamarellou et al., 2008; Cai et al., 2012). In addition, *A. baumannii* has a significant ability to produce biofilms on various surfaces (Espinal et al., 2012; Longo et al., 2014), which not only increases the potential of *A. baumannii* for nosocomial spread, but also contributes to its resistance to antibiotics and virulence (Lee et al., 2008; Rao et al., 2008). Thus, there is a critical need to explore novel strategies for treating *A. baumannii* infections.

Traditionally, plants have served as a source of novel drugs for treating a variety of diseases in humans (Cowan, 1999). A variety of plant-derived compounds possessing antimicrobial properties against a wide range of microorganisms have been documented (Kon and Rai, 2012; Upadhyay et al., 2014). The antimicrobial effects of four components of ginger against MDR strains of *A. baumannii* has been reported (Wang et al., 2010). Similarly, the essential oil from coriander was found to exert either synergistic or additive effects with antibiotics such as tetracycline, chloramphenicol, ciprofloxacin, gentamicin, piperacillin, and cefoperazone against *A. baumannii* (Duarte et al., 2012). Recently, aqueous extract of kiwi (*Actinidia deliciosa*) and clove (*Syzygium aromaticum*) were found to exert anti-biofilm activity against *A. baumannii* (Tiwari et al., 2017). Similarly, it was shown that plant extract from *Aegle marmelos* and imipenem had synergistic effect against carbapenem resistant strain of *Acinetobacter baumannii* (Tiwari et al., 2016). In another study, the essential oil from *Origanum vulgare* possessed potent antimicrobial activity against MDR *A. baumannii* (Saghi et al., 2015). In addition, previous research from our laboratory indicated that several plant-derived antimicrobials (PDAs), including *trans*-cinnamaldehyde (TC), an ingredient in cinnamon, eugenol (present in clove) and carvacrol and thymol obtained from oregano oil and oil of thyme, respectively, decreased antibiotic resistance in MDR *S*. Typhimurium DT 104 (Johny et al., 2010). These investigators observed that TC reduced DT 104’s resistance to five antibiotics, where thymol and carvacrol decreased resistance to three antibiotics.

The β-lactam group of antibiotics are the most commonly prescribed antibiotics for the treatment of bacterial infections worldwide (Pitout et al., 1997; Thakuria and Lahon, 2013). *A. baumannii* is capable of producing β-lactamases that can hydrolyze the β-lactam ring of penicillins, cephalosporins, and carbapenems, thereby conferring resistance to these antibiotics. Therefore, this study investigated the efficacy of TC and eugenol (EG) in increasing the sensitivity of *A. baumannii* to seven β-lactam antibiotics. In addition, the effect of these PDAs on genes conferring resistance to β-lactam antibiotics in *A. baumannii* was determined.

## Materials and Methods

### *A. baumannii* Cultures and Growth Conditions

Two clinical isolates of *A. baumannii*, namely 251847 (International Health Management Associates, IL), and 17978 (ATCC; fatal meningitis isolate) were used in the study. All bacteriological media used in the study, except Leeds MDR *Acinetobacter* agar, were purchased from Difco (Becton Dickinson, Sparks, MD, United States). Leeds MDR agar was procured from Hardy Diagnostics (Santa Maria, CA, United States). The bacterial isolates were cultured separately overnight in 10 ml tryptic soy broth (TSB), followed by streaking on Leeds MDR *Acinetobacter* agar plates and incubation at 37°C for 24 h. An individual colony from Leeds MDR *Acinetobacter* agar was sub-cultured twice in 10 ml of TSB at 37°C for 24 h with agitation to reach ∼8 log_10_ CFU/ml. The cultures were sedimented by centrifugation (3,700 × *g*, 15 min, 4°C), and the pellet was washed twice and re-suspended in sterile phosphate buffered saline (PBS; pH 7.2). The cultures were then diluted appropriately in PBS to obtain ∼5 to 6 log_10_ CFU/ml to be used as the inoculum. The bacterial population in the inoculum was confirmed by plating on tryptic soy agar (TSA) with incubation at 37°C for 24 h.

### Plant-Derived Antimicrobials (PDAs) and Chemicals

*Trans*-cinnamaldehyde (≥98%; TC, *trans*-3-phenyl-2-propenal), eugenol (≥98%; EG,4-allyl-2-methoxyphenol), 1-*N*-phenylnaphthylamine (NPN), EDTA, ethidium bromide (EtBr), carbonyl cyanide m-chlorophenylhydrazone (CCP) and pyronin Y were purchased from Sigma-Aldrich (St. Louis, MO, United States).

### Determination of Sub-inhibitory Concentration (SIC) and Minimum Inhibitory Concentration (MIC) of PDAs Against *A. baumannii*

The sub-inhibitory concentration (SIC) and minimum inhibitory concentration (MIC) of TC and EG against *A. baumannii* were determined as previously reported (Johny et al., 2010; Amalaradjou and Venkitanarayanan, 2011) TSB (10 ml) tubes containing 0.75–7.5 μM (TC) and 0.61–6.1 μM (EG) in increments of 0.375 μM (TC) and 0.305 μM (EG were inoculated separately with *A. baumannii* at ∼ 6 log_10_ CFU/ml, and incubated at 37°C for 24 h. Tubes without any added PDAs served as controls. After incubation, the samples were serially diluted (1:10) in PBS, plated on TSA, and incubated at 37°C for 24 h before counting the colonies. The highest concentration of TC or EG that did not inhibit *A. baumannii* growth after 24 h of incubation was selected as the SIC, while the lowest concentration of the antimicrobial that inhibited visible growth of the bacteria after 24 h incubation was taken as the MIC of that treatment. The experiment was done in duplicates and repeated three times.

### Effect of PDAs on Antibiotic Resistance in *A. baumannii*

The β-lactam antibiotics tested in the current study included Ampicillin, Meropenem, Methicillin, Penicillin, Aztreonam, Amoxicillin, and Piperacillin (Sigma-Aldrich). Previously published MIC of each aforementioned antibiotic against *A. baumannii* was used in the study (Vashist et al., 2011; Morfin-Otero and Dowzicky, 2012; Malone and Kwon, 2013) (**Table 1**). To determine the effect of combination of PDAs and antibiotics on *A. baumannii*, the MIC of each antibiotic and that of TC/EG were added to 10 ml TSB containing *A. baumannii* (∼ 6 log_10_ CFU/ml), and incubated at 37°C for 24 h (Johny et al., 2010). The bacterial counts were determined after broth dilution assay and surface plating of appropriate dilutions on TSA. The treatments included only *A. baumannii* (positive control), *A. baumannii* + antibiotic, *A. baumannii* + PDA and *A. baumannii* + antibiotic + PDA. In addition, suitable controls, including *A. baumannii* + diluent (ethanol) and *A. baumannii* + ethanol + antibiotic were also included. Duplicate samples were included for each treatment, and the experiment was replicated three times.

**Table 1:** MIC of antibiotics used in testing against *A. baumannii.*

Antibiotics	MIC (μg/ml)
Ampicillin	64
Meropenem	32
Methicillin	256
Penicillin	256
Aztreonam	256
Amoxicillin	64
Piperacillin	128


### Efflux Pump Inhibition Assay

To study the effect of TC and EG on inhibiting the action of efflux pumps in *A. baumannii*, EtBr and pyronin Y efflux assays were performed according to a published protocol (Chusri et al., 2009). Overnight cultures of *A. baumannii* were washed twice and resuspended in PBS containing 0.4% glucose to an OD_600_ of ∼0.5. The bacterial suspension was added with the MIC of TC/EG or CCP (positive control, 100 μM), and incubated at 37°C for 5 h. *A. baumannii* suspension in PBS + 0.4% glucose served as control. After incubation, 200 μl of the treatments/control was separately transferred to a 96-well microtiter plate, followed by addition of EtBr (Sigma) to a final concentration of 4 mg/l, and the fluorescence was measured at excitation 530 nm and emission 645 nm. The assay was repeated with pyronin Y (Sigma) at a final concentration of 5 mg/l at 530 nm and emission 645 nm. The experiment was repeated three times with duplicates for *A. baumannii* 17978 and *A. baumannii* 251847.

### Outer Membrane Permeabilization Assay

To study the effect of TC and EG on the outer membrane of *A. baumannii*, NPN uptake assay was performed using a published protocol (Chusri et al., 2009). Overnight *A. baumannii* 17978 and *A. baumannii* 251847 cultures were separately washed and resuspended in 5 mM HEPES buffer to an OD_600_ ∼ 0.5. Aliquots of 100 μl of *A. baumannii* suspension were transferred to a microtiter plate along with the MIC of TC/EG or EDTA 1 mM/HEPES buffer. This was followed by addition of 40 μM of NPN to make the total volume to 200 μl, the fluorescence was measured within 3 min at excitation 355 nm and emission 460 nm, and continuously recorded for 3 h every 10 min. The experiment was repeated three times with duplicates in each treatment.

### Effect of PDAs on Antibiotic Resistance Gene Expression in *A. baumannii*

#### RNA Extraction and cDNA Synthesi*s*

To study the effect of TC and EG on genes associated with resistance to β-lactam antibiotics in *A. baumannii*, bacterial cultures were grown separately with or without the SIC of TC/EG at 37°C in TSB to mid-log phase. *A. baumannii* grown with either antibiotic alone or PDA alone were also included as controls. After incubation, the cultures were subjected to centrifugation (12,000 × g, 15 min, 4°C) and the resultant pellet was added with 0.5 ml of RNAse free, sterile water and 1 ml of RNA protect reagent (Qiagen, Valencia, CA, United States). The total RNA from each sample was extracted using the RNeasy mini kit (Qiagen), and the manufacturer’s instructions were followed in estimating the total RNA using Nanodrop (Thermo Fisher Scientific, Waltham, MA, United States). Super-script II reverse transcriptase kit (Invitrogen, Carlsbad, CA, United States) was used for cDNA synthesis, and the resultant cDNA was used as a template for RT-qPCR. The amplified product was detected using SYBR Green reagents.

#### Real-Time Quantitative PCR (RT-qPCR)

The antibiotic resistance genes of *A. baumannii* assayed for expression analysis included efflux pump genes, namely *adeA, adeB, adeC*; β-lactam resistance gene, *blaP*; and the multidrug resistance protein gene, *mdrp*. Primer Express software^®^ (Applied Biosystems, Foster city, CA, United States) was used for designing the primers specific for the genes and for the endogenous control (16S rRNA). The primers were designed from *A. baumannii* AB0057 genome (CP001182.1) published in the NCBI database (Adams et al., 2008) and their sequences are provided in **Table 2**. The custom synthesized primers were obtained from Integrated DNA Technologies (Foster City, CA, United States). RT-qPCR was performed with StepOnePlus^TM^ Real Time PCR system (Applied Biosystems) using the SYBR green assay (Applied Biosystems) under custom thermal cycling conditions with the normalized RNA as the template (Bookout and Mangelsdorf, 2003). Duplicate samples were analyzed and standardized against 16S rRNA gene expression. The relative changes in mRNA expression levels were determined using comparative threshold cycle (CT) method (2^-ΔΔ*C*_T_^) between the control and the treatments.

**Table 2:** List of primers used in detecting *A. baumannii* antibiotic resistance genes.

Gene	Sequence (5’→3’)	Function
*adeA* (F)	TGACCGACCAATGCACCTT	Efflux pump
(R)	GCAACAGTTCGAGCGCCTAT	
*adeB* (F)	CCGATGACGTATCGAAGTTAGGA	Efflux pump
(R)	CCGATGACGTATCGAAGTTAGGA	
*adeC* (F)	ACGGCCCCAGAAGTCTAGTTC	Efflux pump
(R)	CGATTAACCCCAATAACCCAGTT	
*blaP* (F)	ACACTAGGAGAAGCCATGAAGCTT	Beta-lactam resistance Antibiotics


(R)	GCATGAGATCAAGACCGATAC G	
*mdrp* (F)	GTACGGCTTCTAGACCCACCATTTT	Multiple drug resistance protein


(R)	ACAAAGAGCCGTGCACAGTTT	
rRNA-16S (F)	TCGCTAGTAATCGCGGATCA CGCTGGCGGC	Endogenous control
(R)	GACGGGCGGTGTGTACAAG	


*F, forward; R, reverse.*

### Statistical Analysis

A completely randomized design with a factorial treatment structure was used. The factors included two *A. baumannii* strains, seven antibiotics, and two PDAs. The data were analyzed using the PROC GENMOD procedure of Statistical Analysis Software (SAS ver. 9.4; SAS Institute Inc., Cary, NC, United States). Least square means were considered significant at *P* < 0.05. The data comparisons for gene expression study were made using Students *t*-test. The difference was considered significant at *P* < 0.05.

## Results

**FIGURE 1:**
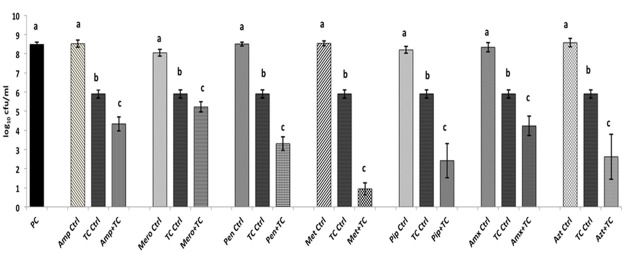
Effect of TC in combination with β-lactam antibiotics in *A. baumannii* 17978. Bars with different superscripts differ from each other within a cluster (*P* < 0.05). *A. baumannii* 17978 was grown with each β-lactam antibiotic either alone or in combination with TC. Bacteria not exposed to any treatments (PC) and bacteria exposed to only TC (TC Ctrl) served as controls for the experiment.

**FIGURE 2:**
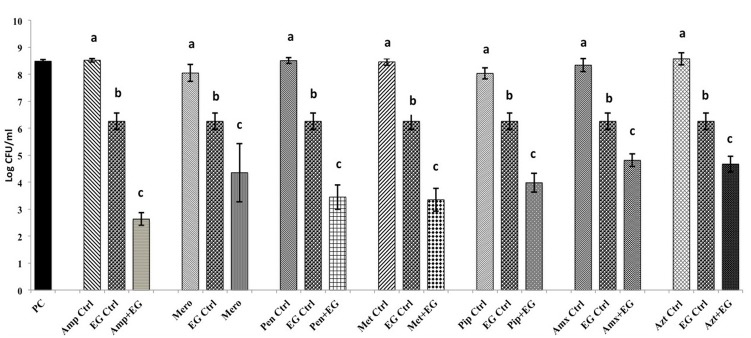
Effect of EG in combination with β-lactam antibiotics in *A. baumannii* 17978. Bars with different superscripts differ from each other within a cluster (*P* < 0.05). *A. baumannii* 17978 was grown with each β-lactam antibiotic either alone or in combination with EG. Bacteria not exposed to any treatments (PC) and bacteria exposed to only EG (EG Ctrl) served as controls for the experiment.

The SICs of TC and EG against *A. baumannii* were found to be 1.1 mM (0.015%) and 1.8 mM (0.03%), respectively, while the MIC was 4 mM (0.05% TC and 0.065% EG) for both PDAs. Although a previously reported MIC of each antibiotic against *A. baumannii* was used in the study, both *A. baumannii* isolates grew ∼ by 2.0 log_10_ CFU/ml after 24 h (antibiotic control), as observed in **Figures 1**, **2** and Supplementary Figures 1, 2. In the presence of the MIC of TC, the bacterial count after 24 h did not change significantly from the inoculation of level of 6.0 log_10_ CFU/ml (**Figure 1**) as expected. However, when *A. baumannii* was grown in the presence of each antibiotic and TC, its growth after 24 h was significantly decreased in comparison to that in the positive control, antibiotic control and TC control (*P* < 0.05). In both *A. baumannii* isolates, the greatest sensitivity was observed to methicillin and lowest sensitivity was seen against ampicillin and meropenem (**Figure 1** and Supplementary Figure 1). Similarly, the sensitivity of both *A. baumannii* isolates to all seven antibiotics was significantly increased in the presence of EG, as indicated by the reduced growth after 24 h (*P* < 0.05) (**Figure 2** and Supplementary Figure 2). In *A. baumannii* 17978, the greatest sensitivity was observed against ampicillin (**Figure 2**), whereas *A. baumannii* 251847 was maximally sensitive to amoxicillin (Supplementary Figure 2).

**FIGURE 3:**
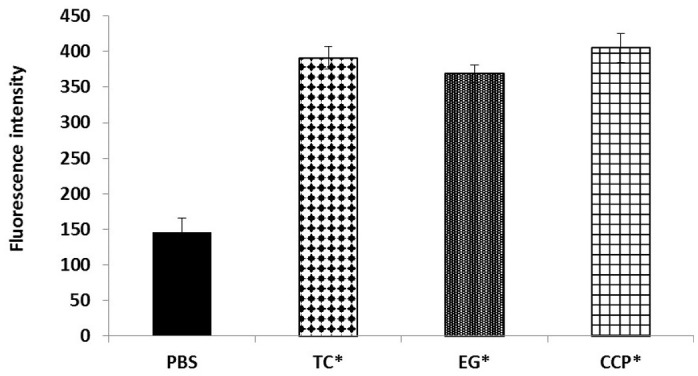
Intracellular accumulation of ethidium bromide (EtBr) in *A. baumannii* ATCC 17978 after treatment with MIC of TC and EG, as measured by fluorescence intensity. *A. baumannii* ATCC 17978 was added with MIC of TC/EG or CCP (100 μM) and incubated for 5 h at 37°C. Fluorescence (excitation 530 nm and emission 645 nm) was measured after addition of EtBr (4 mg/l). Treatments with ^∗^ are significantly different from PBS (control) (*P* < 0.05).

**FIGURE 4:**
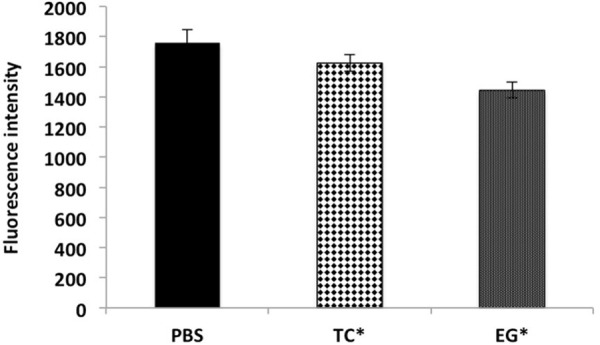
Intracellular accumulation of pyronin Y in *A. baumannii* ATCC 17978 after treatment with MIC of TC and EG, as measured by fluorescence intensity. *A. baumannii* ATCC 17978 was added with MIC of TC/EG and incubated for 5 h at 37°C. Fluorescence (excitation 530 nm and emission 645 nm) was measured after addition of pyronin Y (5 mg/l). Treatments with ^∗^ are significantly different from PBS (control) (*P* < 0.05).

**FIGURE 5:**
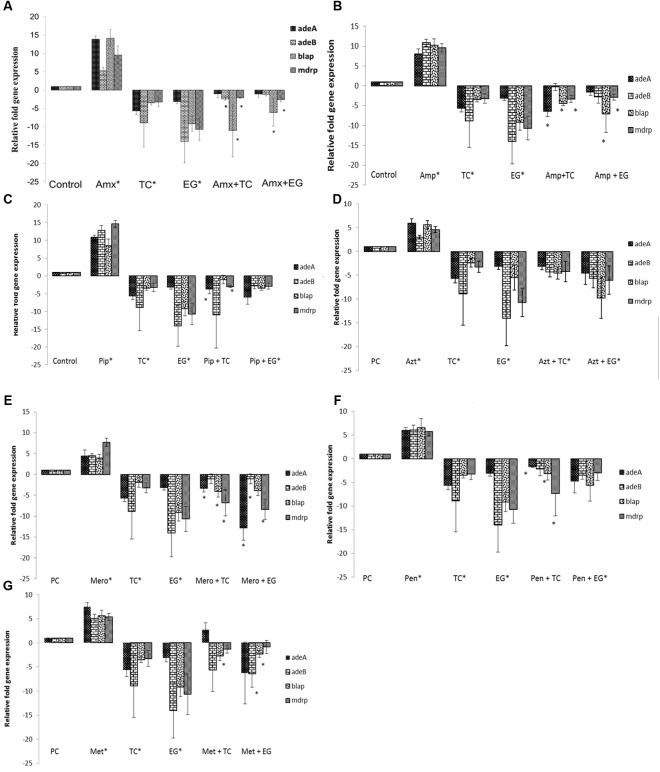
Effect of TC and EG on antibiotic resistance genes in *A. baumannii* 17978. **(A)** *A. baumannii* 17978 was grown with the SIC of TC/EG either alone or in combination with amoxicillin, and RT-qPCR was done to test the effect of the treatments on major antibiotic genes in *A. baumannii.* Bacteria exposed to amoxicillin alone and bacteria not exposed to any treatments served as controls. Bars with ^∗^ are significantly different from control (*P* < 0.05). **(B)** *A. baumannii* 17978 was treated with SICs of TC/EG either alone or in combination with ampicillin and RT-qPCR was done to test the effect of the treatments on major antibiotic genes in *A. baumannii.* Bacteria exposed to ampicillin alone and bacteria not exposed to any treatments served as controls. Bars with ^∗^ are significantly different from control (*P* < 0.05). **(C)** *A. baumannii* 17978 was treated with SICs of TC/EG either alone or in combination with piperacillin and RT-qPCR was done to test the effect of the treatments on major antibiotic genes in *A. baumannii.* Bacteria exposed to piperacillin alone and bacteria not exposed to any treatments served as controls. Bars with ^∗^ are significantly different from control (*P* < 0.05). **(D)** *A. baumannii* 17978 was treated with SICs of TC/EG either alone or in combination with aztreonam and RT-qPCR was done to test the effect of the treatments on major antibiotic genes in *A. baumannii.* Bacteria exposed to aztreonam alone and bacteria not exposed to any treatments served as controls. Bars with ^∗^ are significantly different from control (*P* < 0.05). **(E)** *A. baumannii* 17978 was treated with SICs of TC/EG either alone or in combination with meropenem and RT-qPCR was done to test the effect of the treatments on major antibiotic genes in *A. baumannii.* Bacteria exposed to meropenem alone and bacteria not exposed to any treatments served as controls. Bars with ^∗^ are significantly different from control (*P* < 0.05). **(F)** *A. baumannii* 17978 was treated with SICs of TC/EG either alone or in combination with penicillin and RT-qPCR was done to test the effect of the treatments on major antibiotic genes in *A. baumannii.* Bacteria exposed to penicillin alone and bacteria not exposed to any treatments served as controls. Bars with ^∗^ are significantly different from control (*P* < 0.05). **(G)** *A. baumannii* 17978 was treated with SICs of TC/EG either alone or in combination with methicillin and RT-qPCR was done to test the effect of the treatments on major antibiotic genes in *A. baumannii.* Bacteria exposed to methicillin alone and bacteria not exposed to any treatments served as controls. Bars with ^∗^ are significantly different from control (*P* < 0.05).

The results of the efflux pump inhibition assay using EtBr and pyronin Y in *A. baumannii* ATCC 17978 and *A. baumannii* 251847 are presented in **Figures 3**, **4** and Supplementary Figures 3, 4. EtBr and pyronin Y are known substrates for AdeABC and AdeIJK efflux pumps, respectively (Magnet et al., 2001; Xing et al., 2014). The MIC of TC and EG resulted in an increased accumulation of EtBr inside bacterial cells, as indicated by an increase in fluorescence compared to the PBS control (*P* < 0.05) (**Figures 5A–G**). CCP, an efflux pump inhibitor used as a positive control (Magnet et al., 2001) also resulted in an increase in fluorescence indicating suppression of efflux pump in *A. baumannii*. However, a similar increase in the fluorescence was not observed for pyronin Y compared to the PBS control (**Figure 4** and Supplementary Figure 4). The results of NPN uptake assay in *A. baumannii* ATCC 17978 and *A. baumannii* 251847 are presented in Supplementary Figures 5, 6, respectively. Neither TC nor EG increased NPN uptake, while the EDTA control did show an increase in fluorescence. Further, the samples treated with TC or EG demonstrated a decrease in fluorescence over time (data not shown).

The effect of seven antibiotics and two PDAs and their combination on the expression of various antibiotic resistance genes in *A. baumannii* 17978 is depicted in **Figures 5A–G**. It was observed that compared to control, the expression of all tested genes was up-regulated (*P* < 0.05) following *A. baumannii* growth in the presence of the antibiotics. However, TC significantly down-regulated the expression of major genes conferring resistance to β-lactam antibiotics compared to control (*P* < 0.05). The genes encoding efflux pump *adeA* and *adeB* were down regulated by ∼6- and 9-fold, respectively. Moreover, the expression of genes encoding β-lactamase (*blaP*) and the multiple drug resistance protein (*mdrp*) was decreased by ∼3-fold (*P* < 0.05). Similar to the results observed with TC, all antibiotic resistance genes were down-regulated on exposure to EG compared to control (*P* < 0.05). The expression of genes encoding efflux pumps, *adeA* and *adeB* were down-expressed by 3- and 14-fold, respectively. Similarly, *blaP* and *mdrp* were also down-regulated in both strains (*P* < 0.05). The combination of TC or EG with the antibiotics also resulted in a down-regulation of majority of the tested genes (*P* < 0.05). Among the combinations of TC or EG with the seven antibiotics, the combination containing aztreonam resulted in a significant down-regulation of all the tested genes compared to control (**Figure 5D**). The combination of EG with piperacillin (**Figure 5C**) or penicillin (**Figure 5F**) also significantly reduced the expression of all the antibiotic resistance genes (*P* < 0.05).

## Discussion

Rapid emergence of antibiotic resistance in pathogenic microorganisms, especially to multiple antibiotics has ignited research efforts to discover novel antibiotics and develop effective derivatives of currently available antibiotics. However, no promising antibiotics are under development, and the rapidity and complexity of resistance development in pathogens have further exacerbated the situation. In light of this, a potential viable approach was explored to reduce bacterial antibiotic resistance in the development of inhibitors of resistance mechanisms in bacteria (Renau et al., 1999; Walsh and Fanning, 2008). This strategy involves the co-administration of an antibiotic with an “inhibitor,” which counteracts bacterial resistance mechanism(s), thereby rendering the resistant pathogen sensitive to the drug. The advantage of this approach is that it makes it possible to continue the use of current antibiotics, for which in-depth pharmacological and toxicological data are already available. In this regard, PDAs represent a potential natural group of “inhibitors” of bacterial antibiotic resistance mechanisms.

Abundant literature exists on the antimicrobial properties of a variety of plant compounds against a wide range of microorganisms (Burt, 2004; Upadhyay et al., 2014). However, only a handful of studies have addressed their effect on bacterial antibiotic resistance, especially in Gram negative bacteria (Gallucci et al., 2006; Chusri et al., 2009; Johny et al., 2010; Ilić et al., 2014). In the current study, both TC and EG enhanced the sensitivity of *A. baumannii* to all seven β-lactam antibiotics tested (**Figures 1**, **2** and Supplementary Figures 1, 2). This is evident from the significant reductions in bacterial counts observed in the samples containing PDA and antibiotics as compared to that in the treatments containing each PDA or antibiotic alone. A checkerboard assay testing a wide range of concentrations of two antimicrobials is commonly used for determining their combinatorial effects on a bacterium (Jayamani et al., 2017; Tharmalingam et al., 2018). However, since our preliminary studies revealed that concentrations of TC and EG below the MIC were not significantly effective in increasing *A. baumannii* sensitivity to antibiotics, we did not use a checkerboard assay, but instead tested the combinatorial effect of MIC of plant molecules and antibiotics on the pathogen.

A recent study also reported synergistic effect of *Eucalyptus camaldulensis* with polymyxin B against MDR *Acinetobacter baumannii* (Knezevic et al., 2016). In another study, Enrofloxacin and cinnamon were observed to possess synergistic effect against *Salmonella enterica* (Solarte et al., 2017). Further, extracts of medicinal plant *Holarrhena antidysenterica* with Conessine increased susceptibility of extensively drug resistant *A. baumannii* to novobiocin and rifampicin (Siriyong et al., 2016). Additionally, no cytotoxic effects on human epithelial cells at the tested concentrations were reported previously with TC and EG (Amalaradjou et al., 2010; Karumathil et al., 2016). Moreover our previous studies revealed that in-feed supplementation of TC and EG in broiler chicks (Kollanoor-Johny et al., 2012) and TC in mice (Narayanan et al., 2017) for 10 days did not result in any toxicity.

*A. baumannii* has been reported to exhibit resistance to β-lactam antibiotics through several mechanisms, including the production of β-lactamases, changes in penicillin-binding proteins (PBPs), altering the structure and number of porin proteins, decreased membrane permeability, and by use of efflux pumps that exit antibiotics out of the bacterial cell (Vila et al., 2007; Manchanda et al., 2010; Bonnin et al., 2013; Tang et al., 2014). Moreover, the presence of efflux pumps and MDR proteins in *A. baumannii* contribute significantly to both intrinsic and acquired resistance to antibiotics (Lomovskaya and Bostian, 2006). *A. baumannii* genome encodes a wide array of multidrug efflux systems, including AdeABC, a resistance-nodulation-division (RND) family-type pump (Damier-Piolle et al., 2008; Wieczorek et al., 2008; Yoon et al., 2013; Sun et al., 2014). The substrates for this pump include beta-lactams, aminoglycosides, erythromycin, chloramphenicol, tetracycline, fluoroquinolone, trimethoprim, and EtBr (Magnet et al., 2001; Higgins et al., 2004; Heritier et al., 2005; Peleg et al., 2007). The three component structures of AdeABC include the inner membrane fusion protein (AdeA), transmembrane component (AdeB) and an outer membrane protein (AdeC), with the inactivation of *adeB* resulting in the loss of pump function and multidrug resistance (Magnet et al., 2001).

In order to determine if TC or EG exerted an inhibitory effect on the aforementioned efflux pumps in *A. baumannii*, an efflux pump inhibition assay was performed with EtBr and pyronin Y along with CCP, a documented efflux pump inhibitor (Magnet et al., 2001; Chusri et al., 2009). EtBr and pyronin Y are known substrates for AdeABC and AdeIJK efflux pumps, respectively (Magnet et al., 2001; Damier-Piolle et al., 2008; Chusri et al., 2009; Xing et al., 2014). Both TC and EG resulted in the inhibition of AdeABC efflux pump in the two tested *A. baumannii* strains, as evident from the increase in fluorescence in the treated samples compared to PBS control (**Figure 3** and Supplementary Figure 3). However, the PDAs did not exert any inhibitory effect on the action of AdeIJK efflux pump in *A. baumannii*, as evident from the lack of difference in fluorescence units between PDA-treated and PBS control samples (**Figure 4** and Supplementary Figure 4). Similarly, the results from the NPN uptake assay revealed that TC and EG did not increase *A. baumannii*’s outer membrane permeability, as seen from Supplementary Figures 5, 6, where no increase in fluorescence was observed in PDA-treated *A. baumannii* as against the samples treated with EDTA, a known outer membrane permeabilizer in Gram-negative bacteria (Helander and Mattila-Sandholm, 2000; Alakomi et al., 2006). These results indicate that TC and EG increased the sensitivity of *A. baumannii* to the tested antibiotics at least in part by inhibiting the efflux pump, AdeABC.

For ascertaining if TC or EG exerted an inhibitory effect on any of the antibiotic resistance genes conferring resistance to β-lactam antibiotics in *A. baumannii*, we performed a RT-qPCR on mRNA extracted from *A. baumannii* following growth in the presence and absence of the PDAs. The results from the RT-qPCR revealed that TC and EG in combination with or without each antibiotic significantly down-regulated the expression of the majority of genes that confer resistance to β-lactam antibiotics (**Figures 5A–G**). Among the various genes screened, those encoding efflux pumps, *adeA* and *adeB* were maximally down-regulated by both PDAs. These results concur with the results from the EtBr efflux pump inhibition assay, and suggest that TC and EG enhanced the efficacy of the seven β-lactam antibiotics against *A. baumannii* by thwarting the various resistance mechanisms, especially those involving the efflux pumps.

## Conclusion

The results of this study suggest the potential use of TC and EG in conjunction with the currently available β-lactam antibiotics for the treatment for MDR *A. baumannii* infections and could lead to development of new treatment options with reduced antibiotic dosage. However, efficacy studies in suitable animal models are warranted before recommending their clinical usage.

## Author Contributions

KV is the corresponding author and primary contact during manuscript submission, review, and publication process. The work was done under his supervision as the principal investigator. He significantly contributed to the design, drafting, revisions, and interpretation of data. The manuscript is being submitted with his final approval for publication. MN is the submitting author. DK and AK-J designed the study. DK and JG conducted the experiments. DK and MN prepared the manuscript for submission.

## Conflict of Interest Statement

The authors declare that the research was conducted in the absence of any commercial or financial relationships that could be construed as a potential conflict of interest.
