# Supplementary material for: Trans-Cinnamaldehyde and Eugenol Increase Acinetobacter baumannii Sensitivity to Beta-Lactam Antibiotics
Source: Front Microbiol. 2018 May 23;9:1011. doi: 10.3389/fmicb.2018.01011 (PMC5974060; doi:10.3389/fmicb.2018.01011)
Supplement: Supplementary file 1 [file Data_Sheet_1.DOCX]

***Trans*-Cinnamaldehyde and Eugenol Increase *Acinetobacter baumannii* Sensitivity to Beta-Lactam Antibiotics**

Deepti Prasad Karumathil^1^, James Gaffney^2^, Meera Surendran Nair^1^, Anup Kollanoor-Johny^3^, and Kumar Venkitanarayanan^1*^

^1^ Department of Animal Science, University of Connecticut, Storrs, CT, USA.

^2^ College of Veterinary Medicine, Cornell University, Ithaca, NY, USA.

^3^ Department of Animal Science, University of Minnesota, Saint Paul, MN, USA.

Running title: Effect of *Trans*-cinnamaldehyde and Eugenol on decreasing *A. baumannii* resistance to β-lactam antibiotics.

**^*^**Correspondence: Dr. Kumar Venkitanarayanan, [kumar.venkitanarayanan@uconn.edu](mailto:kumar.venkitanarayanan@uconn.edu) Phone: 860-486-0947, Fax: 860-486-4375.

Supplementary Material:

Supplementary Figures

Sup. Fig 1. Effect of TC in combination with β-lactam antibiotics in *A. baumannii* 251847. Bars with different superscripts differ from each other within a cluster (P<0.05). *A. baumannii* 251847 was grown with each β-lactam antibiotic either alone or in combination with TC. Bacteria not exposed to any treatments (PC) and bacteria exposed to only TC (TC Ctrl) served as controls for the experiment.

Sup. Fig 2. Effect of EG in combination with β-lactam antibiotics in *A. baumannii* 251847. Bars with different superscripts differ from each other within a cluster (P < 0.05). *A. baumannii* 251847 was grown with each β-lactam antibiotic either alone or in combination with EG. Bacteria not exposed to any treatments (PC) and bacteria exposed to only EG (EG Ctrl) served as controls for the experiment.

Sup. Fig 3. Intracellular accumulation of ethidium bromide (EtBr) in *A. baumannii* 251847 after treatment with MIC of TC and EG, as measured by fluorescence intensity. *A. baumannii*251847 was added with MIC of TC/EG or CCCP (100 μM) and incubated for 5 h at 37^o^C. Fluorescence (excitation 530 nm and emission 645 nm) was measured after addition of EtBr (4 mg/l). Treatments with * are significantly different from PBS (control) (P <0.05)

Sup. Fig 4. Intracellular accumulation of pyronin Y in *A. baumannii* 251847 after treatment with MIC of TC and EG, as measured by fluorescence intensity. *A. baumannii* 251847 was added with MIC of TC/EG and incubated for 5 h at 37^o^C. Fluorescence (excitation 530 nm and emission 645 nm) was measured after addition of pyronin Y (5 mg/l). Treatments with * are significantly different from PBS (control) (P <0.05).

Sup. Fig 5. Effect of TC and EG on *A. baumannii* 17978 outer membrane, as measured by fluorescence intensity. HEPES buffer (5.0 mM) was used as a control. *A. baumannii*ATCC 17978 was added with MIC of TC/EG or EDTA (1 mM) and incubated for 5 h at 37^o^C. Fluorescence (excitation 530 nm and emission 645 nm) was measured after addition of NPN (40 μM). Treatments with * are significantly different from HEPES (control) (P <0.05).

Sup. Fig 6. Effect of TC and EG on *A. baumannii* 251847 outer membrane, as measured by fluorescence intensity. HEPES buffer (5.0 mM) was used as a control. *A. baumannii* 251847 was added with MIC of TC/EG or EDTA (1 mM) and incubated for 5 h at 37^o^C. Fluorescence (excitation 530 nm and emission 645 nm) was measured after addition of NPN (40 μM). Treatments with * are significantly different from HEPES (control) (P <0.05).
