# Supplementary figures and images for: Trans-Cinnamaldehyde and Eugenol Increase Acinetobacter baumannii Sensitivity to Beta-Lactam Antibiotics
Source: Front Microbiol. 2018 May 23;9:1011. doi: 10.3389/fmicb.2018.01011 (PMC5974060; doi:10.3389/fmicb.2018.01011)

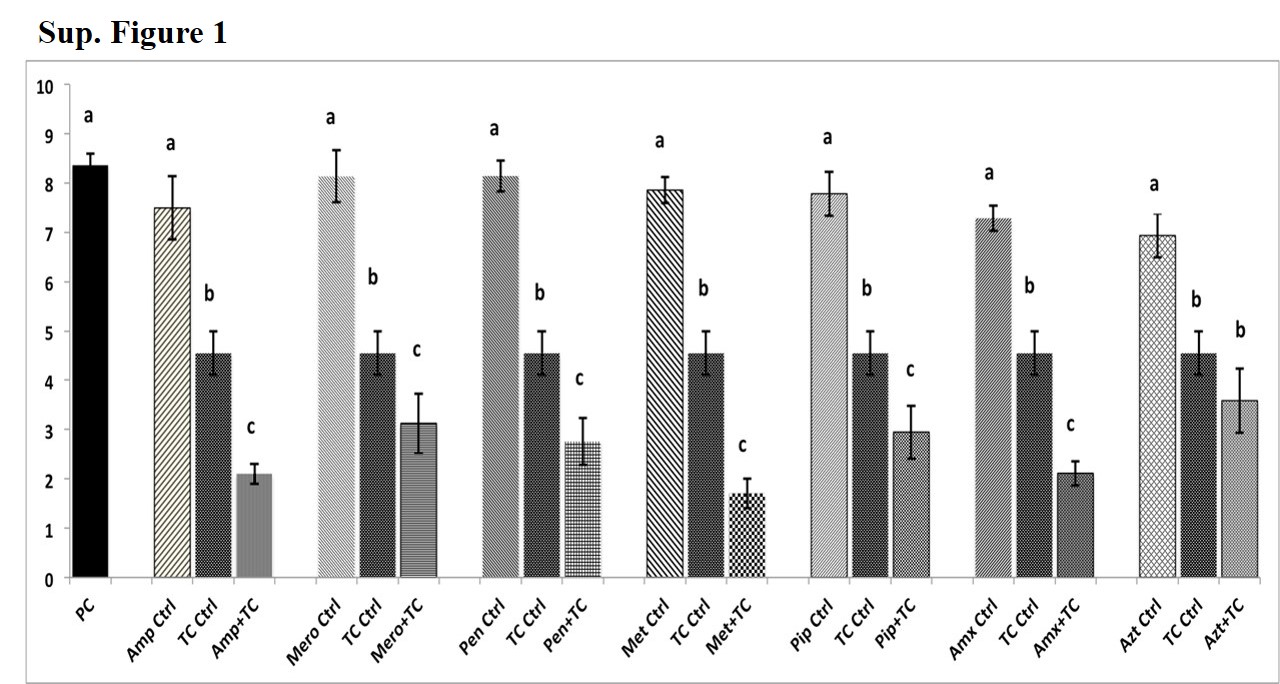

Supplement: Supplementary file 2 [file Image_1.JPEG]

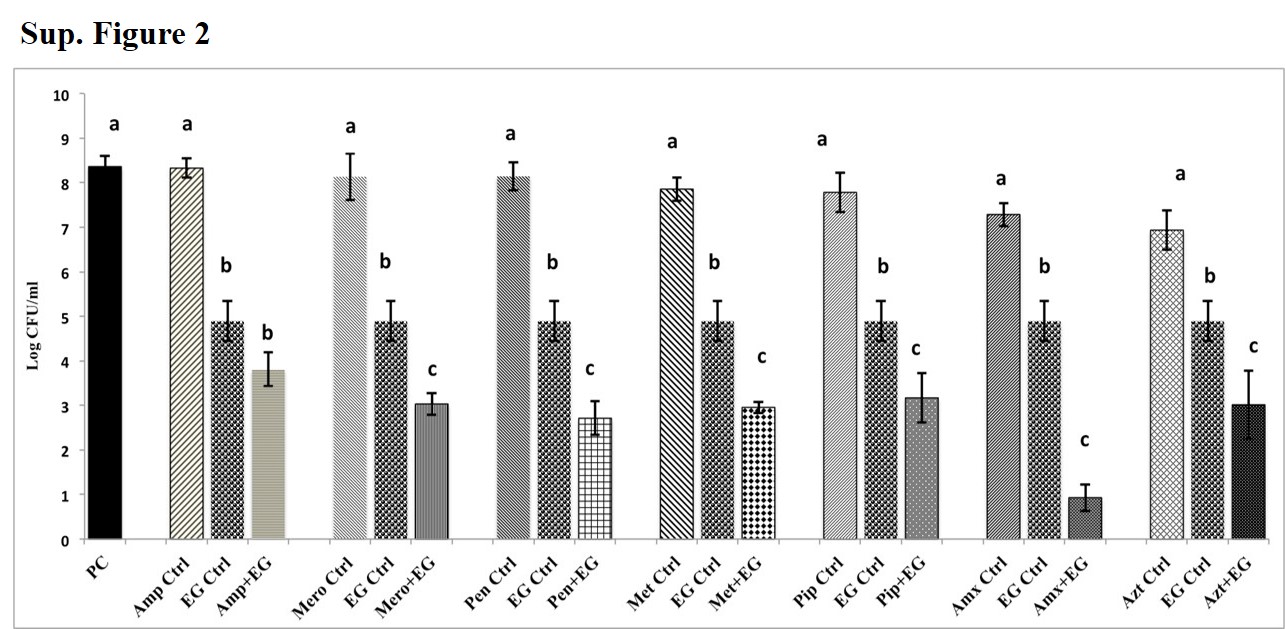

Supplement: Supplementary file 3 [file Image_2.JPEG]

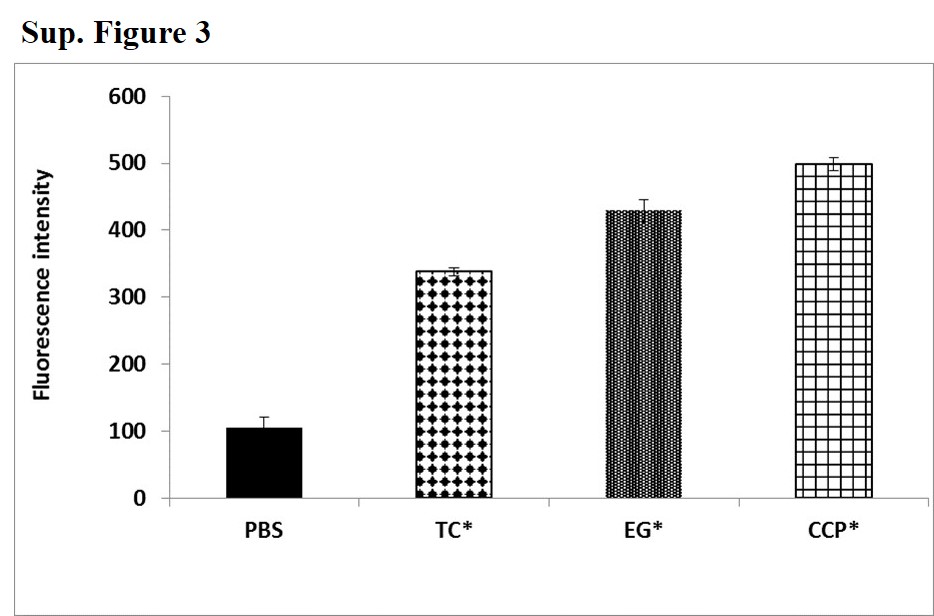

Supplement: Supplementary file 4 [file Image_3.JPEG]

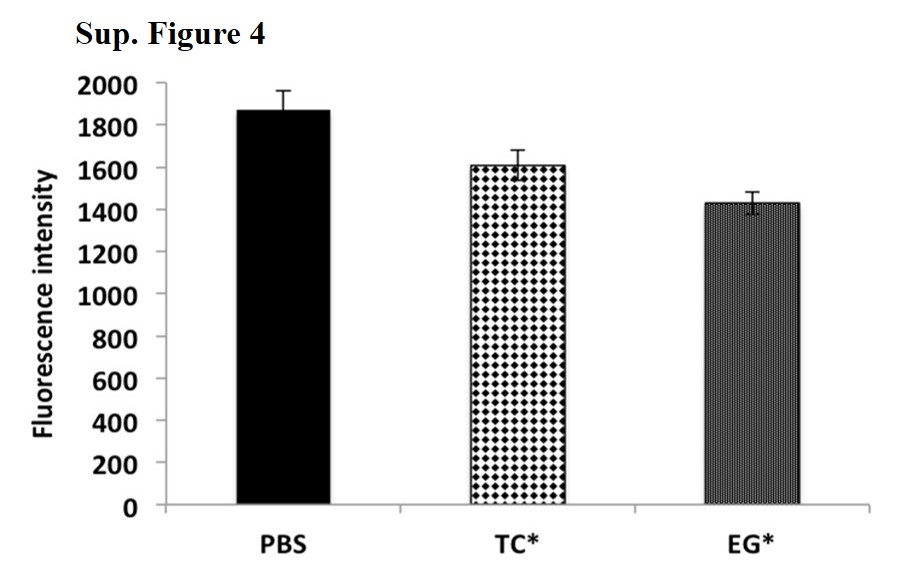

Supplement: Supplementary file 5 [file Image_4.JPEG]

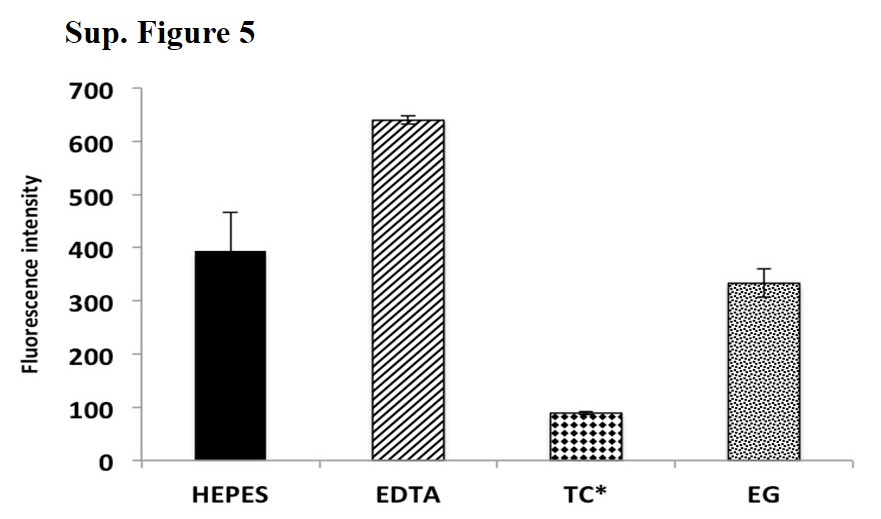

Supplement: Supplementary file 6 [file Image_5.JPEG]

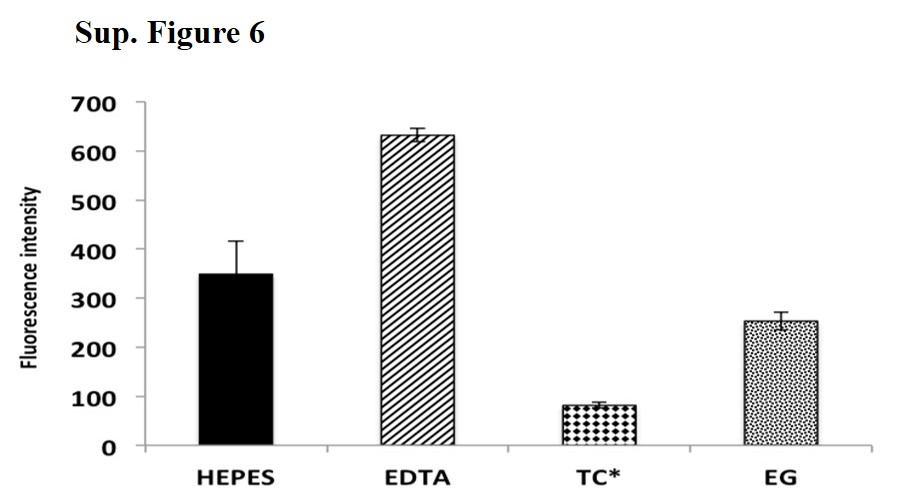

Supplement: Supplementary file 7 [file Image_6.JPEG]
